# Implant impression accuracy of parallel and non-parallel implants: a comparative in-vitro analysis of open and closed tray techniques

**DOI:** 10.1186/s40729-019-0159-5

**Published:** 2019-02-19

**Authors:** Motaz S. Osman, Hassan M. Ziada, Neamat H. Abubakr, Ahmed M. Suliman

**Affiliations:** 1 Department of Oral rehabilitation, Faculty of Dentistry, University of Khartoum, Khartoum, Sudan; 2 Department of Clinical Science, School of Dental Medicine, University of Nevada, Las Vegas, Nevada USA; 3 Department of Clinical Science, School of Dental Medicine, University of Nevada, 1001 Shadow Lane, Suite 248, MS 7415, Las Vegas, Nevada 89106 USA; 4 Department of Oral Maxillofacial Surgery, University of Khartoum, Khartoum, Sudan

**Keywords:** Impression accuracy, Parallel implants, Nonparallel implants, Open tray technique, Closed tray technique

## Abstract

**Background:**

The outcome of the evaluation of impression techniques accuracy may improve the selection criteria for an ideal technique. The aim was to evaluate the accuracy of the open and closed tray techniques for implant impressions, in a partially edentulous maxilla, replaced with a three-unit fixed partial denture, as well as to assess the effect of implants parallelism on accuracy.

**Material and methods:**

This is an experimental in vitro study to evaluate impressions accuracy of a simulated area restored with an implant retained FPD, using the open and closed tray implant impression techniques. The effect of implant position angulation, parallelism, and implant systems (Straumann, SIC Invent, Osstem) was also evaluated. Three custom-made acrylic resin test models were prepared with two parallel and two non-parallel implants, on either side of a maxillary arch. One hundred and ninety-two impressions were made using monophase VPS impression material. Their master casts were obtained and evaluated for the horizontal and vertical discrepancy. The casts were scanned using a model scanner. The distances between the two reference points were measured.

**Results:**

The Straumann and SIC Invent implants showed no statistically significant differences (Mann-Whitney *U* test), regarding accuracy for both the open and closed tray impression techniques (*P* = 0.667 and *P* = 0.472). There were no significant differences for the parallel and non-parallel implants (*P* = 0.323 and *P* = 0.814), respectively, while the Osstem system showed statistically significant differences for both the open and closed tray impression techniques (*P* = 0.035) and between the parallel and non-parallel implants (*P* = 0.045). For the vertical discrepancies, significant differences were detected (chi-square test) between the open and closed tray impression techniques (*P* = 0.037).

**Conclusions:**

Within the limitations of this study, there were generally no significant differences between open and closed, although better results were obtained for the open tray techniques. On the use of the non-parallel implants, the open tray technique provided a better result than the closed tray technique.

## Introduction

A three-dimensionally accurate impression is a pre-requisite for implant restorations since there is no intervening periodontal ligament at the implant-bone interface to compensate for any inaccuracies [1, 2]. Numerous factors impact on implant impression accuracy, including the technique, the materials used, and the number of implants, as well as the parallelism of the implants or abutments. Impression inaccuracies impact negatively on the precision fit of the restoration [1, 3]. Consequently, mechanical complications may arise, such as screw or abutments loosening fracture of the prosthetic components or the implant. Marginal or vertical discrepancies may also develop, increasing plaque accumulation, which may also negatively impact on the soft and hard tissues around the implant [1, 3].

The open and closed tray impression techniques are both advocated, each has advantages and disadvantages. Non-parallel implants may strain the impression during tray removal, due to the significant force required for its withdrawal, which compromises accuracy [1]. Regardless of the technique and the number of implants or parallelism, the use of a verification device would be advisable, to ensure a clinically passive metal framework fit [4].

There is no evidence of the superiority of one impression technique or material over others. Nonetheless, an accurate impression is fundamental, in achieving a passive prosthetic fit and the long-term serviceability. In this regard, Mpikos et al. [5] found neither the open nor the closed tray techniques influenced the accuracy of impressions of multiple implants. However, they found implant parallelism had a significant impact on impression accuracy, particularly in implants with internal connections. Conrad et al. [2] reported that the average angle of error and the magnitude of distortion between the closed and open tray techniques were not significantly different. However, Alexander Hazboun et al. [6] found neither the open or closed tray techniques nor the implant angulations (0, 15, and 30 degrees) had any significant effect on impression accuracy.

It is generally difficult to detect vertical or marginal discrepancies clinically. In this regard, restorations are considered “passive,” if they do not create any static loading within the prosthetic system or bone. Occlusal inconsistency increases the incidence of mechanical complications, i.e., screw loosening and/or prosthesis and implant fracture [1, 2], since unlike natural teeth, osseointegrated implants do not have periodontal ligaments to compensate for any occlusal inconsistencies. Furthermore, superstructure misfits with vertical discrepancies may increase the plaque accumulation, negatively impacting on soft and/or hard tissues around implants. However, such biologic complications on bone and tissues around implants are still controversial [1, 2].

We hypothesize that making an impression in a closed or open technique for two implants in a maxillary Kennedy class III for restoration with an implant retained FPD, regardless of parallelism, would have no impact on accuracy. The aim of this study is, therefore, to evaluate the accuracy of the open and closed tray implant impression techniques, in a Kennedy class III partially edentulous jaws (to be restored with an implant retained FPD), and the effect of implant angulation on impression accuracy.

## Methods and materials

The present in vitro investigation was conducted to evaluate the accuracy of the open and closed tray impression techniques and the effect of parallelism/angulation of two implants for constructing an implant-retained FPD, in a Kennedy class III partially edentulous maxilla.

**Fig. 1:**
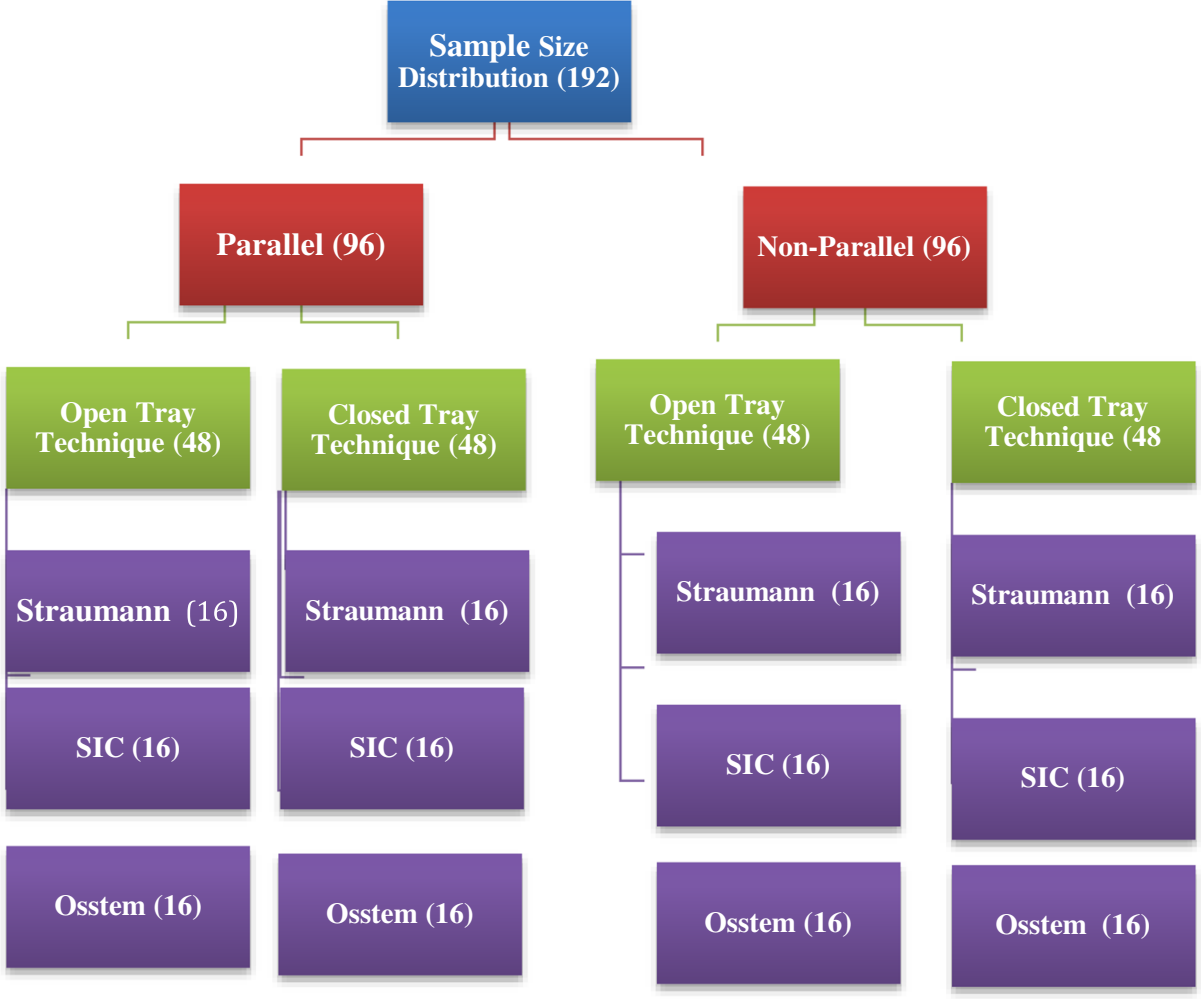
Flowchart of the sample distribution

The sample calculated was based on data from a previous study [7]. A sample that would produce the power for analysis for this study was derived as 13, for each impression technique, for the three-implant systems. It was increased to 16, to allow for potential error during preparation. Accordingly, 192 impressions were made, including 96 parallel and 96 non-parallels, and figure shows the sample distribution (Fig. 1).

Three custom-made acrylic resin models (each model for every implant system) were made from heat cure acrylic resin. These models were constructed to simulate a partially edentulous maxilla, of a Kennedy class III, with missing first premolar, second premolar, and the first permanent molar. Initially, silicon impressions were made of the case cast, and molten wax was poured in the silicon mold, to create three wax modes. These wax models were boiled and packed with heat-polymerized acrylic resin (Lucitone-199 DENTSPLY) to construct three acrylic models, which were designated the “test models”.

**Fig. 2:**
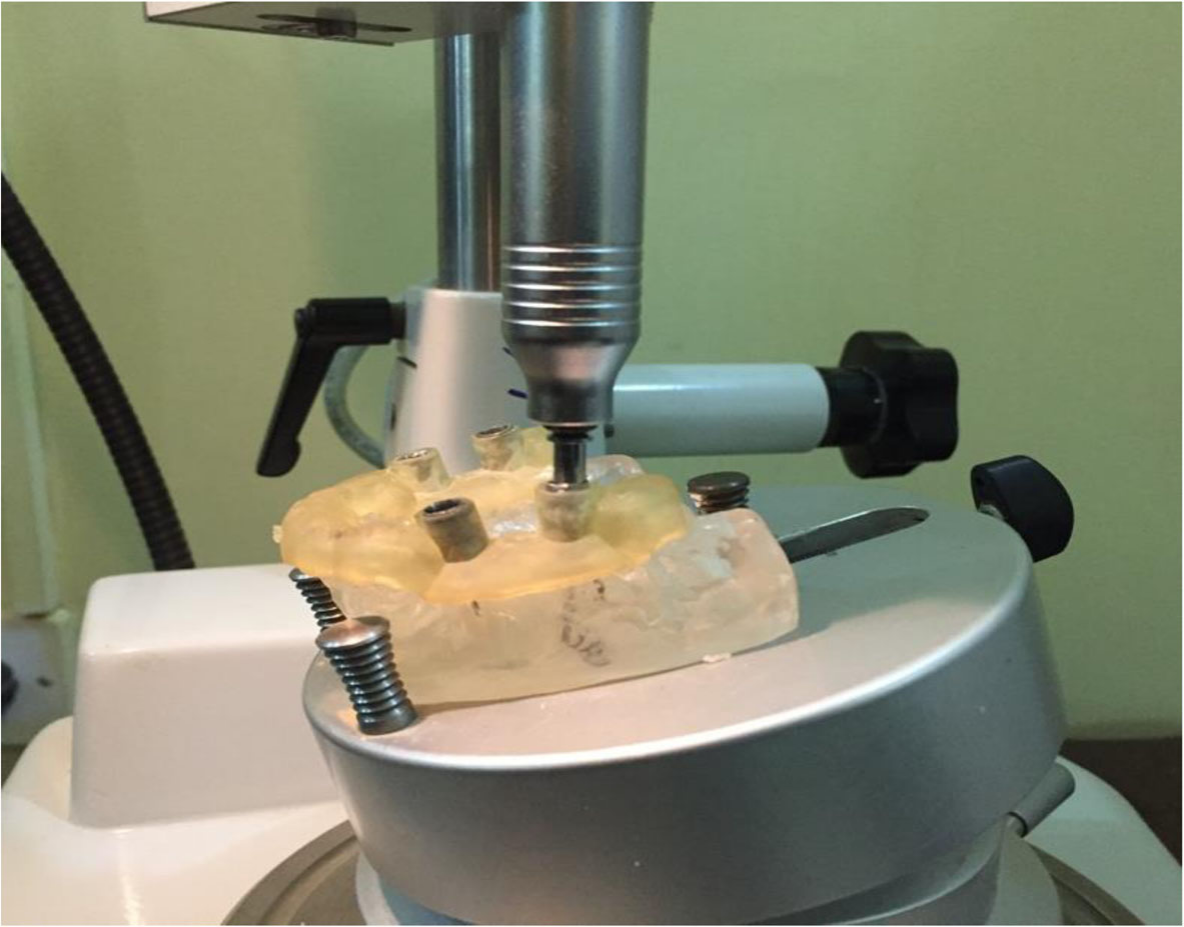
Dental surveyor for the standardization of drilling, angulations, and implant installments

For the standardization of implants positioning and installation, surgical guides were designed with CAD/CAM software and constructed using 3D printing. The implants were then installed in the test models, using a dental surveyor and a milling machine (BEGO Paraskop M Germany) (Fig. 2). Tripoding was first made to ensure reproducible in the positioning of the test models on the surveyor. Four implants were installed in each test model. On the right side, in the first premolar region along a straight axis, while in the first molar region, the implant position was tilted distally and installed in a 15° angulation. On the left side, the two implants were parallel along a straight axis to each other, in the areas of the first premolar and first molars. The surgical guide was then securely positioned on each test model, and implant drilling made through the surgical guide sleeve, using the sequence for each implant system. To make additionally sure that the installed implants were stable within the models, they were cemented using luting adhesive resin cement (Multilink/Ivoclar Vivadent) [8].

The first test model had the Straumann Implant System (Straumann AG, Basel, Switzerland), with implant fixtures diameter of 4.1 mm and a length of 10.0 mm. The second test model had the SIC Invent Implant System (SIC invent Deutschland GmbH, Germany) with an implant fixture diameter of 4.0 mm and a length of 11.5 mm. The third test model had the Osstem Implant System (426-5, Gasan-dong, Geumcheon-gu, Seoul, Korea) with an implant fixture diameter of 4.0 mm and a length of 11.5 mm.

### Horizontal measurements

The three test models were scanned using a high-resolution dental scanner (Activity 885 Smart Optics - Sensortechnik GmbH, Bochum, Germany) [9]. The digital horizontal distances between the two implants on the parallel and non-parallel implants sites were measured from center to center of implant fixtures using the software (exocad-Dental CAD). The distance on the right side between the implants at first premolar site (along axis) and the first molar site (distally angulated, 15°) was assigned as distance 1 (D1), and the distance on the left side between first premolar and first molar (parallel implants) sites were designated as distance 2 (D2). These measurements were recorded as the test model baseline reference measurements. Each test model was then stabilized on the table, the impression copings inserted and secured to their implant fixtures, and the impressions were made (Virtual Monophase vinyl polysiloxane - Ivoclar Vivadent AG.), and repeated for every implant system. The impressions were checked to fulfill the evaluation criteria described by Lee and Gallucci [10]. The criteria include the following:

- There should be an exact imprint and reproduction of the implant areas.
- The impression copings should not be displaced from the impression.
- There should be no voids in the occlusal, buccal, lingual, and interproximal surfaces of the neighboring teeth.
- The impression material should not be separated from the custom tray.

Any impression not meeting any of these criteria was repeated.

The impression copings were then reinserted and secured in their corresponding implant analogs, poured using type IV dental stone (Elite Rock, Zhermack, Italy). The casts were separated after 45 min, according to the manufacturer’s instruction. They were then stored at room temperature for 24 h before the second horizontal measurements made [7]. For these second horizontal distance measurements, the master casts were scanned, similar to the test models (Activity 885 smart optics), and the D1 and D2 were measured using software exocad-Dental CAD. The measurements were recorded and used to compare the horizontal distances measurements between the test models and the cast digital measurements, for every technique and each implant system.

### The vertical evaluation

**Fig. 3:**
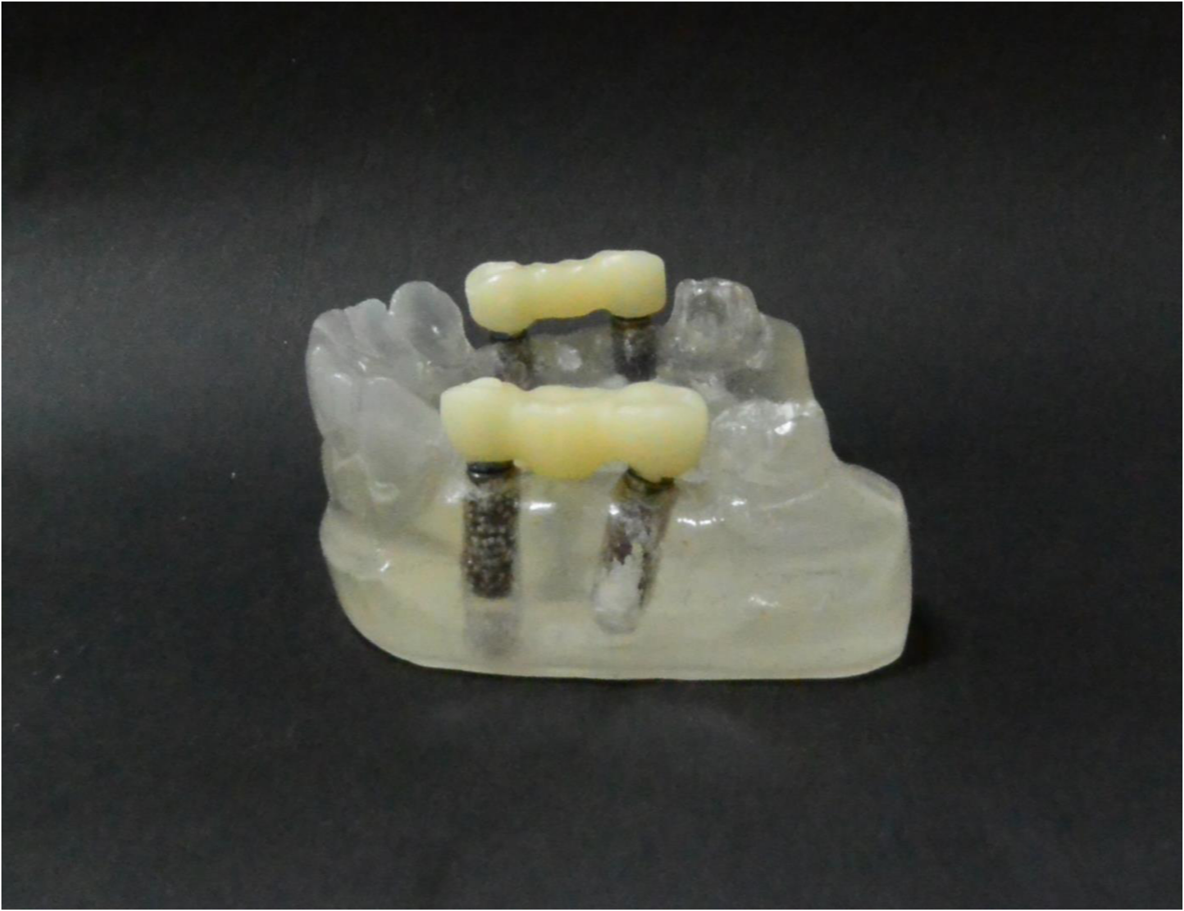
CAD/CAM verification jig

The stone casts were later sectioned to a base of 20 mm, to facilitate placement under a stereomicroscope. Verification jigs were initially fabricated from CAD/CAM acrylic resin blocks (BILKIM PMMA blank for CAD-CAM applications 14 mm -A2 color-Turkey) on the test models (Fig. 3). These verification jigs were made to evaluate vertical discrepancies on the master casts under a stereomicroscope.

**Fig. 4:**
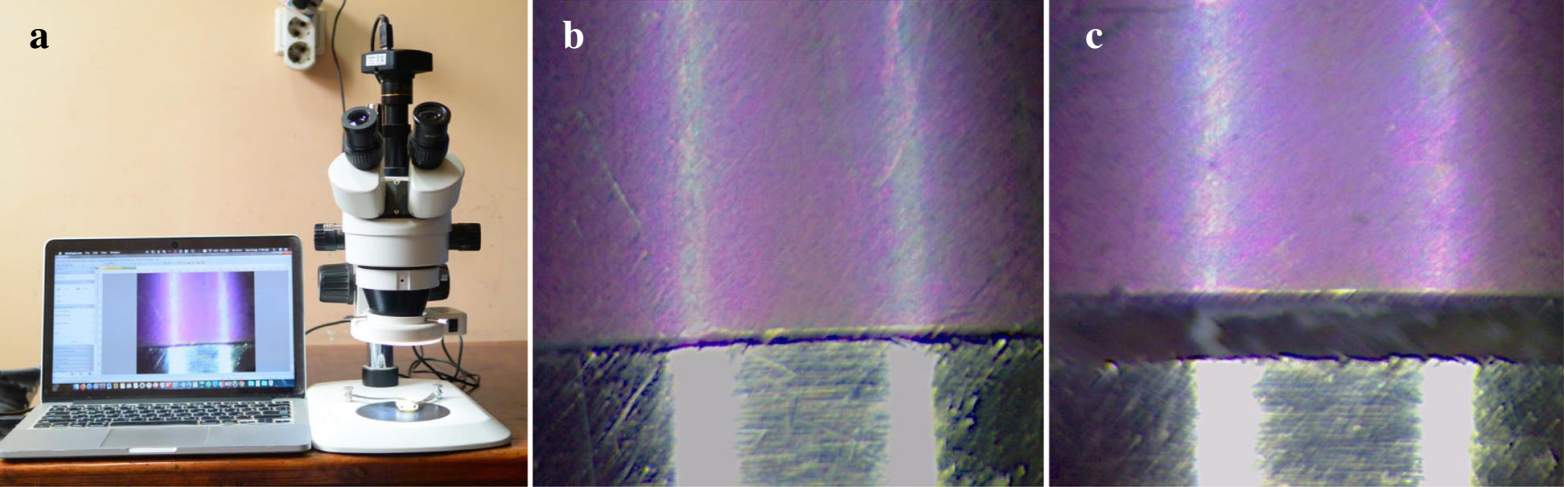
**a** Stereomicroscope used. **b** No vertical/marginal discrepancy. **c** Presence of vertical and marginal discrepancy

Tightening the screws on the laboratory implant analogs, on the sectioned master cast, retained the jigs [11]. The presence or absence of the vertical discrepancies on the sectioned casts was then evaluated under the stereomicroscope (AmScop14370, Myford Road, #150, Irvine, CA 92606 USA) at × 50 magnifications and related data recorded (Fig. 4a–c).

### Statistical analysis

Data were tabulated and statistically analyzed using IBM SPSS Statistics software version 22. The data collected from the three-implant systems were classified and used to compare the effects of implant impression techniques and parallelism. The *p* value was set at *p* ≤ 0.05 and regarded as statistically significant.

## Results

One hundred ninety-two impressions, including 96 parallel and 96 non-parallel implants were made for the three test models with the three implant systems. There were no statistically significant differences (Mann-Whitney *U* test) in impression accuracy between the open and closed tray techniques (*P* = 0.057). For the parallel and non-parallel implants, there were also no statistically significant differences between the two techniques (Mann-Whitney *U* test, *P* = 0.244, *P* = 0.145) (Table 1).

**Table 1:** Accuracy of implant impression technique in the horizontal direction

Variable	Impression technique	*N*	Median	Mean	Standard deviation	*P* value
Impression technique	Open	96	0.0145	0.034	0.042	0.057
	Closed	96	0.0320	0.049	0.032	
Parallel implant	Open	48	0.0140	0.030	0.039	0.244
	Closed	48	0.0170	0.041	0.042	
Non-parallel implant	Open	48	0.0155	0.039	0.044	0.145
	Closed	48	0.0355	0.057	0.060	

Mann-Whitney *U* test, *P* value < 0.05 no significance difference

For the implant system, the Straumann and SIC Invent implants showed no statistically significant differences (Mann-Whitney *U* test), in the open and closed tray impression techniques (*P* = 0.667 and *P* = 0.472, respectively), while the Osstem system showed statistically significant differences between the two impression techniques (*P* = 0.035) (Table 2).

**Table 2:** Accuracy of impression technique for parallel and non-parallel implant systems

Implant systems	Impression technique	*N*	Median	Mean	Standard deviation	*P* value
Straumann	Open	32	0.016	0.036	0.044	0.667
	Closed	32	0.016	0.038	0.041	
SIC	Open	32	0.020	0.037	0.038	0.472
	Closed	32	0.036	0.047	0.045	
Osstem	Open	32	0.011	0.029	0.043	0.035^*^
	Closed	32	0.037	0.062	0.066	
Straumann	Parallel	32	0.016	0.031	0.034	0.323
	Non-parallel	32	0.016	0.044	0.049	
SIC	Parallel	32	0.020	0.040	0.040	0.814
	Non-parallel	32	0.032	0.044	0.043	
Osstem	Parallel	32	0.012	0.035	0.047	0.045^*^
	Non-parallel	32	0.017	0.056	0.066	

^*^Mann-Whitney *U* test, *P* value < 0.05 statistically significant

There was also no statistically significant differences (Mann-Whitney *U* test) in impression accuracy between the parallel and non-parallel implants, for the Straumann and SIC Invent implant systems (*P* = 0.323 and *P* = 0.814, respectively). There were, however, significant differences between the parallel and non-parallel implants for the Osstem implant system (*P* = 0.045*) (Table 2).

For the open tray technique, significant differences were observed (Mann-Whitney *U* test) between parallel and non-parallel implants for the Ostem implant system (*P* value 0.0166*), while no significant differences in this regard for the Straumann (*P* value 0.926) and SIC Invent implant systems (*P* value 0.999) (Table 3).

**Table 3:** Accuracy of open and closed tray impression techniques for both parallel and non-parallel implant systems

Impression technique	Implant system	Angulations	Count	Median	Mean	SD	*P* value
Open tray	Straumann	Parallel	16	0.017	0.028	0.031	0.926
		Non-parallel	16	0.015	0.045	0.054	
	SIC Invent	Parallel	16	0.019	0.040	0.045	0.999
		Non-parallel	16	0.020	0.034	0.030	
	Osstem	Parallel	16	0.008	0.021	0.038	0.0166^*^
		Non-parallel	16	0.016	0.037	0.047	
Closed tray	Strauman	Parallel	16	0.015	0.034	0.037	0.196
		Non-parallel	16	0.016	0.043	0.045	
	SIC Invent	Parallel	16	0.028	0.040	0.036	0.616
		Non-parallel	16	0.041	0.053	0.053	
	Osstem	Parallel	16	0.026	0.049	0.052	0.423
		Non-parallel	16	0.039	0.075	0.078	

^*^Mann-Whitney *U* test, *P* value < 0.05 statistically significant

In the closed tray technique, no significant differences between parallel and non-parallel implants observed for the three implant systems (Mann-Whitney *U* test): Ostem (*P* value 0.423), Straumann (*P* value 0.196), and SIC Invent implant system (*P* value 0.616) (Table 3).

For vertical discrepancies evaluations, significant differences were detected (chi-square test) between the open and closed tray impression techniques (*P* = 0.037*). For the parallel and non-parallel implants evaluations, there were no significant differences between the open and closed tray impression techniques (*P* = 0.112, *P* = 0.135) (Table 4).

**Table 4:** Vertical discrepancy and impression technique accuracy

Variables	Impression technique	Vertical discrepancy		Total	*P* value
		Yes (%)	No (%)		
Impression technique	Open	20 (20.8%)	76 (79.2%)	96	0.037*
	Closed	32 (33.3%)	64 (66.7%)	96	
	Total	52 (27%)	140 (73%)	192	
Parallel implant	Open	8 (16.7%)	40 (83.3%)	48	0.112
	Closed	14 (29.2%)	34 (70.8%)	48	
	Total	22 (23%)	74 (77%)	96	
Non-parallel implant	Open	12 (25%)	36 (75%)	48	0.135
	Closed	18 (37.5%)	30 (62.5%)	48	
	Total	30 (31%)	66 (69%)	96	

Chi-square test, *P* value < 0.05 statistically significant

*The differences between open and closed tray techniques accuracy regarding vertical discrepancy

**Fig. 5:**
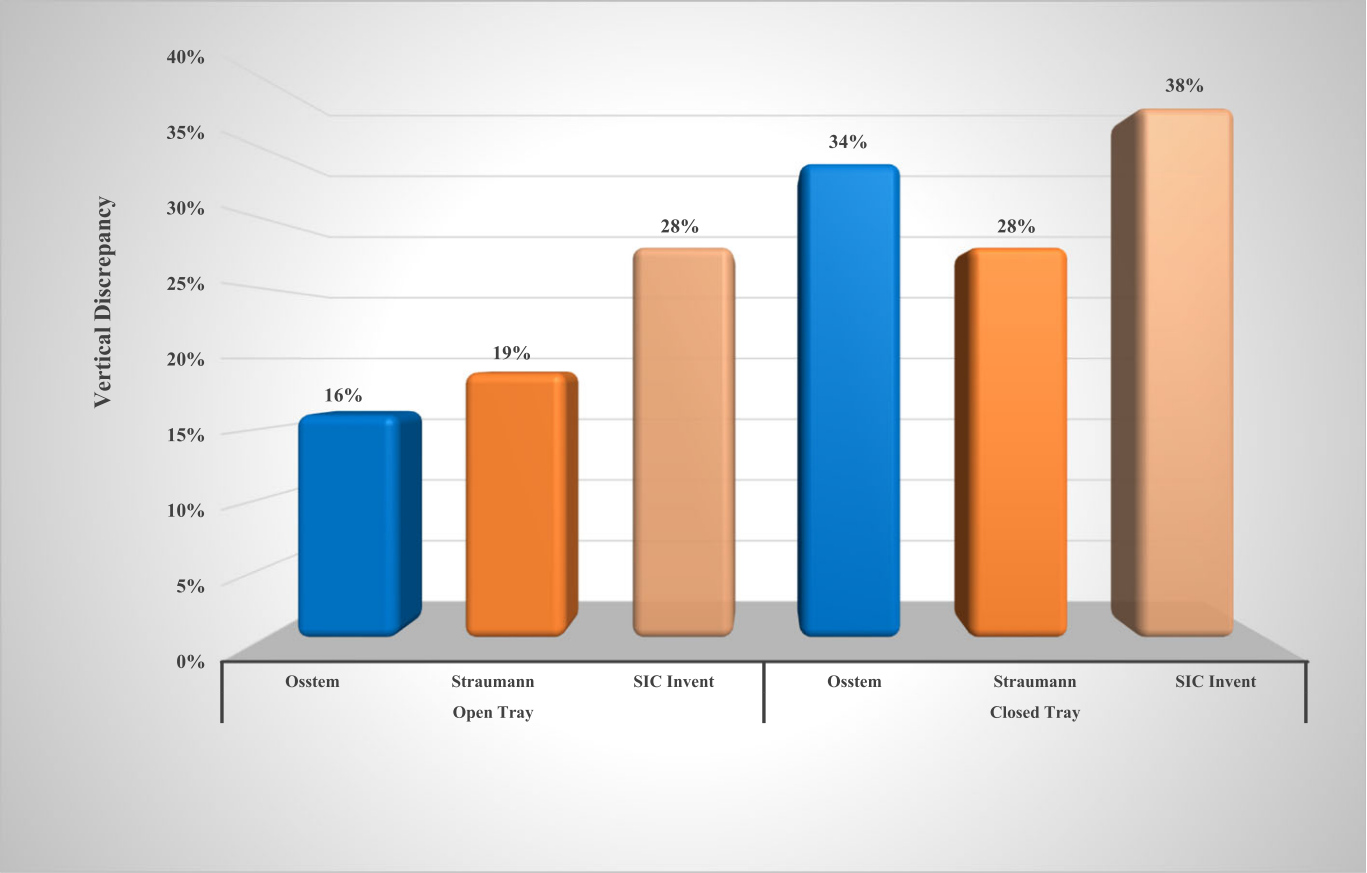
Effect of implant impression techniques on the vertical discrepancy of all implant systems

Regarding the vertical discrepancy for open tray implant impression techniques, the vertical discrepancy occurred in 16% of the Osstem implant system (5 out of 32), in 19% of the Straumann implant system (6 out of 32 sample), and in 28% of the SIC Invent systems (9 out of 32). For the closed tray techniques, the vertical discrepancy in the Osstem implant system occurred 34% (11 out of 32) and 28% of the Straumann implant system (9 out of 32), while in the SIC Invent, it occurred in 38% (12 out of 32) (Fig. 5).

**Fig. 6:**
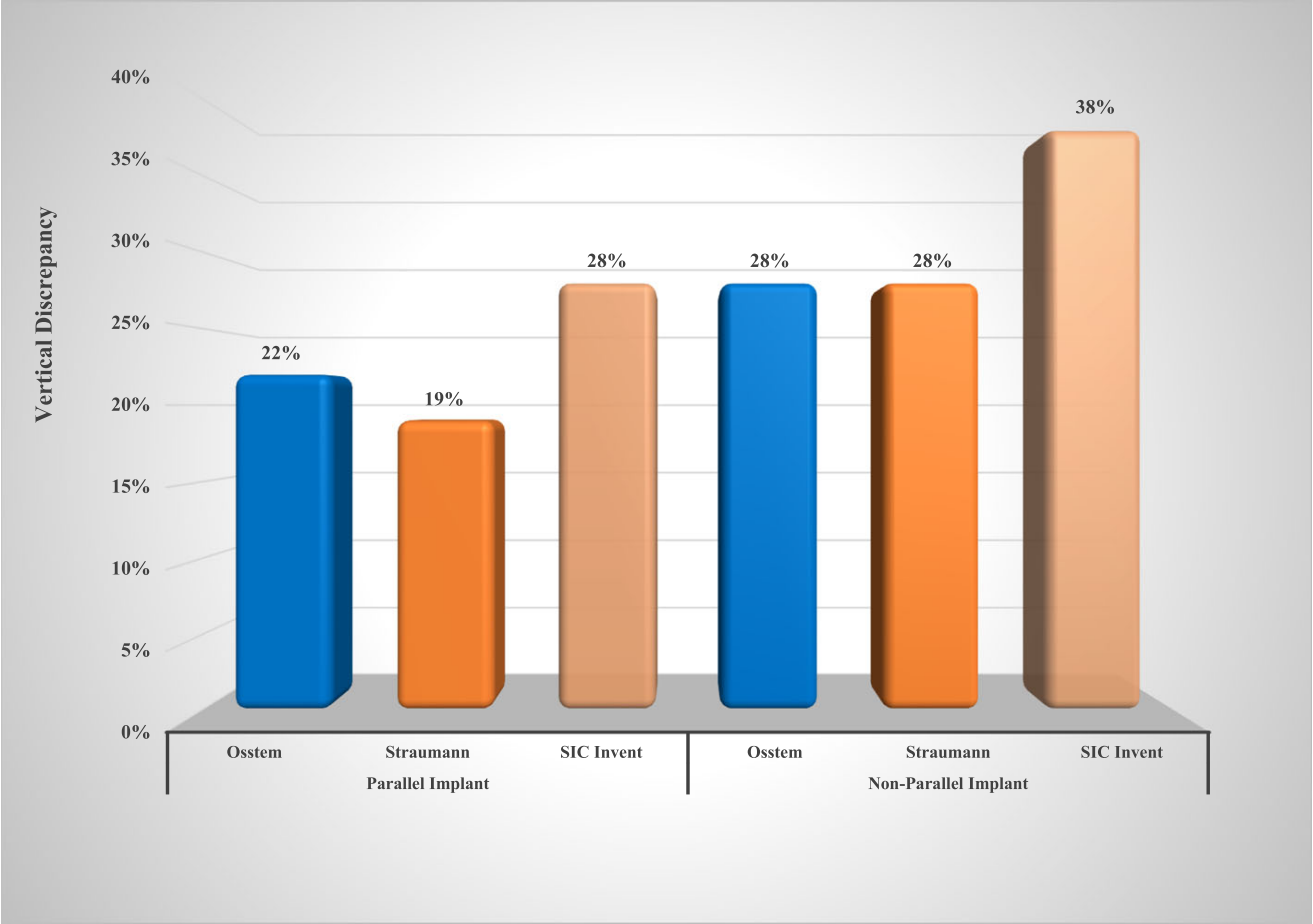
Effect of implant angulation on the vertical discrepancy of all implant systems

For the parallel implants, the vertical discrepancy occurred in 22% (7 out of 32) of the Osstem system and 19% for the Straumann system (6 out of 32), while it was occurred in 28% of SIC Invent (9 out of 32). For the non-parallel implants, the vertical discrepancy occurred in 28% (9 out of 32 samples) of the Osstem system and 28% (9 out of 32) of the Straumann system. While the SIC Invent had the highest percentage, 38% (12 out of 32) (Fig. 6).

Logistic regression estimates were made with vertical discrepancy as a dependent variable, the closed tray impression technique, parallel implants, and the Straumann implant system as reference categories. There was a significant association between vertical discrepancy and the impression technique (open tray vs. closed tray *P* value 0.000). There was no significant association between the three implant systems (Straumann vs. Osstem *P* value 0.065 and Straumann vs. SIC Invent *P* value 0.501). Also, there was no significant association regarding the implants angulation (parallel vs. non-parallel, *P* value 0.864). There was an inverse relationship between the open tray technique and vertical discrepancy (Table 5).

**Table 5:** Logistic regression estimates test

Variable	*B*	S.E.	Wald	Df	*P* value	OR	95% C.I. for (OR)	
							Lower	Upper
Techniques-open	− 1.053	0.298	12.476	1	0.000*	0.349	0.195	0.626
Angulated	− 0.048	0.284	0.029	1	0.864	0.953	0.547	1.661
SIC Invent	− 0.215	0.319	0.453	1	0.501	0.807	0.432	1.507
Osstem	− 0.618	0.335	3.411	1	0.065	0.539	0.280	1.039

^*^Dependent variable: vertical discrepancy

## Discussion

The primary purpose of the implant impression is to transfer the implant/abutment position from the oral cavity to the master cast. The impression material for implants should be rigid enough to hold the impression copings and minimize positional distortion during replica positioning [12]. The polyvinylsiloxane (PVS) and polyether both seem to be the materials of choice for making an accurate impression [13]. In the current study, polyvinylsiloxane (PVS) was used, since it was reported to have superior deformation recovery, higher physical and mechanical properties, less potential of dimensional changes, precise reproduction of details, and desirable modulus of elasticity. It is also easier to remove from undercuts, with less deformation, making it a popular choice in implant dentistry. Several studies used polyvinylsiloxane (PVS) in similar investigations for evaluating implant impressions accuracy [13–17].

In the present experimental analysis, no statistically significant difference was found between the open and closed impression techniques, in agreement with Wenz et al. [15]. The open tray technique was superior and more accurate in studies by Daoudi et al., AlQuran et al., and Elshenawy et al. [18–20]. The present study found that the mean data for the open tray technique were likely to produce more predictable impressions compared to the closed tray technique; however, the differences were not statistically significant. The current study certainly does not concur with the findings of Balouch et al., [21] who reported the closed impression technique to have less dimensional changes.

We used a high-resolution dental scanner (Activity 885 Smart Optics) in this study as an evaluation method, due to its high efficiency and accuracy. It can also be carried out in a shorter time and measures to the third decimal unit, while the calipers measure to two the second decimal unit. Besides, calipers are handheld, while the scanner is fixed, increasing the confidence in accuracy.

The rationale of posterior tilting of a distal implant in this study is that anatomical and esthetic considerations may not always allow parallel positioning of the implants. Such placement would be a valid compromise to bone grafting, maxillary sinus lift, or mandibular nerve displacement, with the added benefit of shorter treatment times, decreased potential morbidity, and reduced cost [22, 23]. The present finding was similar to the study conducted by Alexander Hazboun and colleagues [6], reporting impression techniques (open vs. closed tray) and implant angulation (0, 15, and 30°) had no significant effect on in vitro impression accuracy. Similarly, Conrad et al. [2] found that the angle of error between closed and open tray techniques did not differ significantly since the magnitude of distortion was similar for all combinations of impression techniques, implant angulation, and implant numbers.

Rutkunas et al. [24], showed the open tray technique to be more accurate with highly non-axially oriented implants. Jang et al. [25] found that various implant divergent angles (0, 5, 10, 15, and 20°), particularly those with the internal connection, were more accurately recorded with the open tray technique. It seems that while unfavorable parallelism may be corrected prosthetically, the lack of parallelism still creates a path of removal that may distort the impression material, leading to an inaccurate master cast.

A limitation of this study is the lack of three-dimensional evaluation and analysis. Hence, in this two-dimensional evaluation and analysis, some information may have been lost during the assessment. Also, using computer-aided design/computer-assisted manufacture (CAD/CAM) and three-dimensional (3D) optical digitization may have led to different results. Nevertheless, it still would be considered a simple and a perceptive means of evaluating the accuracy of varying impression techniques [23]. Therefore, further studies should examine the effect of multiple implant positions, with various angulations and depths, and with various impression materials. Another limitation is that being an in vitro study, it is not clear if data from this study would be similar in the clinical setting. The authors are currently analyzing data on the outcome of the two impression techniques in a clinical study.

## Conclusion

Within the limitation of this study, the open and closed tray implant impression techniques showed a similar level of accuracy. For the non-parallel implants, the open tray technique provided a better result than the closed tray technique. The open tray impression technique exhibited the least horizontal and vertical discrepancies for the Straumann, SIC Invent, and Osstem implant systems.

## References

[1] Sorrentino R, Gherlone EF, Calesini G, Zarone F (2010). Effect of implant angulation, connection length, and impression material on the dimensional accuracy of implant impressions: an in vitro comparative study. Clin Implant Dent Relat Res.

[2] Conrad HJ, Pesun IJ, DeLong R, Hodges JS (2007). Accuracy of two impression techniques with angulated implants. J Prosthet Dent.

[3] Lee H, So JS, Hochstedler JL, Ercoli C (2008). The accuracy of implant impressions: a systematic review. J Prosthet Dent.

[4] Lin WS, Harris BT, Metz MJ, Morton D (2014). A technique for verifying and correcting a milled polyurethane definitive cast for nonsegmental implant restoration in an edentulous jaw. J Prosthet Dent.

[5] Mpikos P, Kafantaris N, Tortopidis D, Galanis C, Kaisarlis G, Koidis P (2012). The effect of impression technique and implant angulation on the impression accuracy of external- and internal-connection implants. Int J Oral Maxillofac Implants.

[6] Alexander Hazboun GB, Masri R, Romberg E, Kempler J, Driscoll CF (2015). Effect of implant angulation and impression technique on impressions of NobelActive implants. J Prosthet Dent.

[7] Herbst D, Nel JC, Driessen CH, Becker PJ (2000). Evaluation of impression accuracy for osseointegrated implant supported superstructures. J Prosthet Dent.

[8] Shim JS, Ryu JJ, Shin SW, Lee JY (2015). Effects of implant angulation and impression coping type on the dimensional accuracy of impressions. Implant Dent.

[9] Technical data sheet: Activity 885, Smart Optics Sensortechnik GmbH, Bochum, Germany. https://www.smartoptics.de/en/dental/dental-scan/.

[10] Lee SJ, Gallucci GO (2013). Digital vs. conventional implant impressions: efficiency outcomes. Clin Oral Implants Res.

[11] Papaspyridakos P, Lal K, White GS, Weber HP, Gallucci GO (2011). Effect of splinted and nonsplinted impression techniques on the accuracy of fit of fixed implant prostheses in edentulous patients: a comparative study. Int J Oral Maxillofac Implants.

[12] Gokcen-Rohlig B, Ongul D, Sancakli E, Sermet B (2014). Comparative evaluation of the effects of implant position, impression material, and tray type on implant impression accuracy. Implant Dent.

[13] Prithviraj DR, Pujari M, Garg P, Shruthi D (2011). Accuracy of the implant impression obtained from different impression materials and techniques: review. J Clin Exp Dent.

[14] Sabouhi M, Bajoghli F, Dakhilalian M, Beygi A, Abolhasani M (2016). Effects of impression coping design, impression technique, and dental undercuts on the accuracy of implant impressions assessed by 3-dimensional optical scanning: an in vitro study. Implant Dent.

[15] Wenz HJ, Hertrampf K (2008). Accuracy of impressions and casts using different implant impression techniques in a multi-implant system with an internal hex connection. Int J Oral Maxillofac Implants.

[16] Hamalian TA, Nasr E, Chidiac JJ (2011). Impression materials in fixed prosthodontics: influence of choice on clinical procedure. J Prosthodont.

[17] Gallucci GO, Papaspyridakos P, Ashy LM, Kim GE, Brady NJ, Weber HP (2011). Clinical accuracy outcomes of closed-tray and open-tray implant impression techniques for partially edentulous patients. Int J Prosthodont.

[18] Daoudi MF, Setchell DJ, Searson LJ (2004). An evaluation of three implant level impression techniques for single tooth implant. Eur J Prosthodont Restor Dent.

[19] Al Quran FA, Rashdan BA, Zomar AA, Weiner S (2012). Passive fit and accuracy of three dental implant impression techniques. Quintessence Int.

[20] Elshenawy EA, Alam-Eldein AM, Abd Elfatah FA (2018). Cast accuracy obtained from different impression techniques at different implant angulations (in vitro study). Int J. Implant Dent.

[21] Balouch F, Jalalian E, Nikkheslat M, Ghavamian R, Toopchi S, Jallalian F (2013). Comparison of dimensional accuracy between open-tray and closed-tray implant impression technique in 15 degrees angled implants. J Dent.

[22] Papaspyridakos P, Chen CJ, Gallucci GO, Doukoudakis A, Weber HP, Chronopoulos V (2014). Accuracy of implant impressions for partially and completely edentulous patients: a systematic review. Int J Oral Maxillofac Implants.

[23] Kim JH, Kim KR, Kim S (2015). Critical appraisal of implant impression accuracies: a systematic review. J Prosthet Dent.

[24] Rutkunas V, Sveikata K, Savickas R (2012). Effects of implant angulation, material selection, and impression technique on impression accuracy: a preliminary laboratory study. Int J Prosthodont.

[25] Jang HK, Kim S, Shim JS, Lee KW, Moon HS (2011). Accuracy of impressions for internal-connection implant prostheses with various divergent angles. Int J Oral Maxillofac Implants.

